# In situ loop-mediated isothermal amplification (LAMP) for identification of *Plasmodium* species in wide-range thin blood smears

**DOI:** 10.1186/s12936-018-2381-7

**Published:** 2018-06-19

**Authors:** Muneaki Hashimoto, Hirokazu Sakamoto, Yusuke Ido, Masato Tanaka, Shouki Yatsushiro, Kazuaki Kajimoto, Masatoshi Kataoka

**Affiliations:** 1 Health Research Institute, National Institute of Advanced Industrial Science and Technology (AIST), 2217-14, Hayashi-cho, Takamatsu, Kagawa 761-0301 Japan; 2 Present Address: Department of Biochemistry and Molecular Biology, Graduate School and Faculty of Medicine, The University of Tokyo, 7-3-1, Hongo, Bunkyo-ku, Tokyo, 113-0033 Japan

**Keywords:** Malaria, *Plasmodium*, Diagnosis, Parasite species, In situ LAMP, Thin blood smears

## Abstract

**Background:**

Five species of *Plasmodium* are known to infect humans. For proper treatment of malaria, accurate identification of the parasite species is crucial. The current gold standard for malaria diagnosis is microscopic examination of Giemsa-stained blood smears. Since the parasite species are identified by microscopists who manually search for the parasite-infected red blood cells (RBCs), misdiagnosis due to human error tends to occur in case of low parasitaemia or mixed infection. Then, molecular methods, such as polymerase chain reaction or loop-mediated isothermal amplification (LAMP), are required for conclusive identification of the parasite species. However, since molecular methods are highly sensitive, false-positive results tend to occur due to contamination (carry over) or the target gene products may be detected even after clearance of the parasites from the patient’s blood. Therefore, accurate detection of parasites themselves by microscopic examination is essential for the definitive diagnosis. Thus, the method of in situ LAMP for the parasites was developed.

**Results:**

Red blood cell suspensions, including cultured *Plasmodium falciparum,* strain 3D7, infected-RBCs, were dispersed on cyclic olefin copolymer (COC) plate surfaces rendered hydrophilic by reactive ion-etching treatment using a SAMCO RIE system (hydrophilic-treated), followed by standing for 10 min to allow the RBCs to settle down on the plate surface. By rinsing the plate with RPMI 1640 medium, monolayers of RBCs formed on almost the entire plate surface. The plate was then dried with a hair drier. The RBCs were fixed with formalin, followed by permeabilization with Triton X-100. Then, amplification of the *P. falciparum 18S rRNA* gene by the LAMP reaction with digoxigenin (DIG)-labelled dUTP and a specific primer set was performed. Infected RBCs as fluorescence-positive cells with anti-DIG antibodies conjugated with fluorescein using fluorescent microscopy could be detected.

**Conclusions:**

The present work shows that the potential of in situ LAMP for the identification of *Plasmodium* species at the single cell level on hydrophilic-treated COC palates, allowing highly sensitive and accurate malaria diagnosis. The findings will improve the efficacy of the gold standard method for malaria diagnosis.

## Background

Malaria is an infectious disease caused by different species of *Plasmodium*, of which, five species are reported to infect humans (i.e., *Plasmodium falciparum*, *Plasmodium vivax*, *Plasmodium malariae*, *Plasmodium ovale*, and *Plasmodium knowlesi*). *Plasmodium* parasitizes red blood cells (RBCs) and disrupts the host cells, resulting in the occurrence of fever and/or anaemia. Since malaria caused by *P. falciparum* is the most serious, with high mortality, accurate and prompt diagnosis is especially important to effective management. Microscopic examination of thick and thin, Giemsa-stained blood films (‘Giemsa microscopy’) has been the gold standard for the diagnosis of malaria [1]. Although Giemsa microscopy with thick blood films is useful to detect the parasites in patients with low parasitaemia, this technique has some limitations. Diagnosis by this method tends to underestimate the infection rate [2] and is not recommended for identification of the parasite species [3]. Microscopic examination of Giemsa-stained thin blood smears is a better method for accurate parasitaemia estimation and identification of the parasite species compared to the microscopic examination of thick blood films. Parasitemia generally indicates the severity of the malaria infection and accurate identification of the species enables an appropriate choice of anti-malarial medicine and better treatment of the disease.

However, at a time, only a small area of a thin blood smear provides a monolayer of RBCs suitable for microscopic evaluation, thereby limiting the number of cells that can be examined for parasitaemia estimation [4]. Recently, a method that allows microscopic analysis of Giemsa-stained RBCs spread in a monolayer over the entire surface of hydrophilic-treated plastic plates was established to improve Giemsa microscopy with thin blood smears [5]. Microscopists examine differential-diagnostic details, such as infected RBCs, parasite size and shape, or characteristic dots in the RBC stroma [6]. Giemsa microscopy does not always show typical *Plasmodium*-infected RBCs (iRBCs). Therefore, identification of the parasite species requires examination of as many iRBCs as possible. Misdiagnosis particularly occurs in case of low parasitaemia and also in case of mixed infection where the abundance of a particular parasite species may dominate the presence of other species [7, 8]. Further, misdiagnosis also occurs when the quality of the blood smear is poor.

Genetic analysis, such as polymerase chain reaction (PCR), is a very sensitive diagnostic method for the detection and identification of *Plasmodium* species [9, 10]. Recently, Lau et al. reported that loop-mediated isothermal amplification (LAMP), which is more sensitive than PCR with a processing time of less than 60 min at low temperature (~ 65 °C) [11, 12], can be used for the identification of the parasite species [13]. Rapid diagnostic tests (RDTs) based on the immunochromatographic capture procedure using monoclonal antibodies are also used for the identification of the parasite species; however, the possibility of misdiagnosis is a well-known disadvantage of RDTs [14].

One of the advantages of molecular methods, such as PCR or LAMP, is that an accurate diagnosis can be made using the gene-specific primer set. However, false-positive results obtained by PCR analysis after clearance of the parasites from the patients’ bloodstream has been reported [15]. Further, false-positive results tend to occur via molecular methods resulting from contamination (carry over) due to these high sensitivity [16, 17]. Therefore, a definitive diagnosis of malaria should be made by detecting the specific parasite species itself during microscopic examination. Hence, in situ LAMP was developed where gene amplification can be performed in iRBCs on thin blood smears. Development of in situ LAMP for virus-infected cells [18] and bacteria [19, 20] has been reported. Recently, a method that allows the formation of a monolayer over the whole surface of hydrophilic-treated plastic plates were reported [5]. In this study, in situ LAMP was performed in iRBCs on the hydrophilic-treated plates to analyze as many iRBCs as possible on a slide. The identification of malarial parasite at cellular levels using in situ LAMP assay was able to be performed.

## Methods

### Malarial parasite culture and preparation of hydrophilic-treated cyclic olefin copolymer (COC) plate surface

*Plasmodium falciparum* strain 3D7 was cultured, as previously described [21]. Plastic plates (25 × 75 mm) made of COC (Shin-Etsu Polymer Co. Ltd., Tokyo, Japan) were rendered hydrophilic by reactive ion-etching treatment using a SAMCO RIE system (SAMCO Inc., Tokyo, Japan) as previously described [22].

### In situ LAMP

**Fig. 1:**
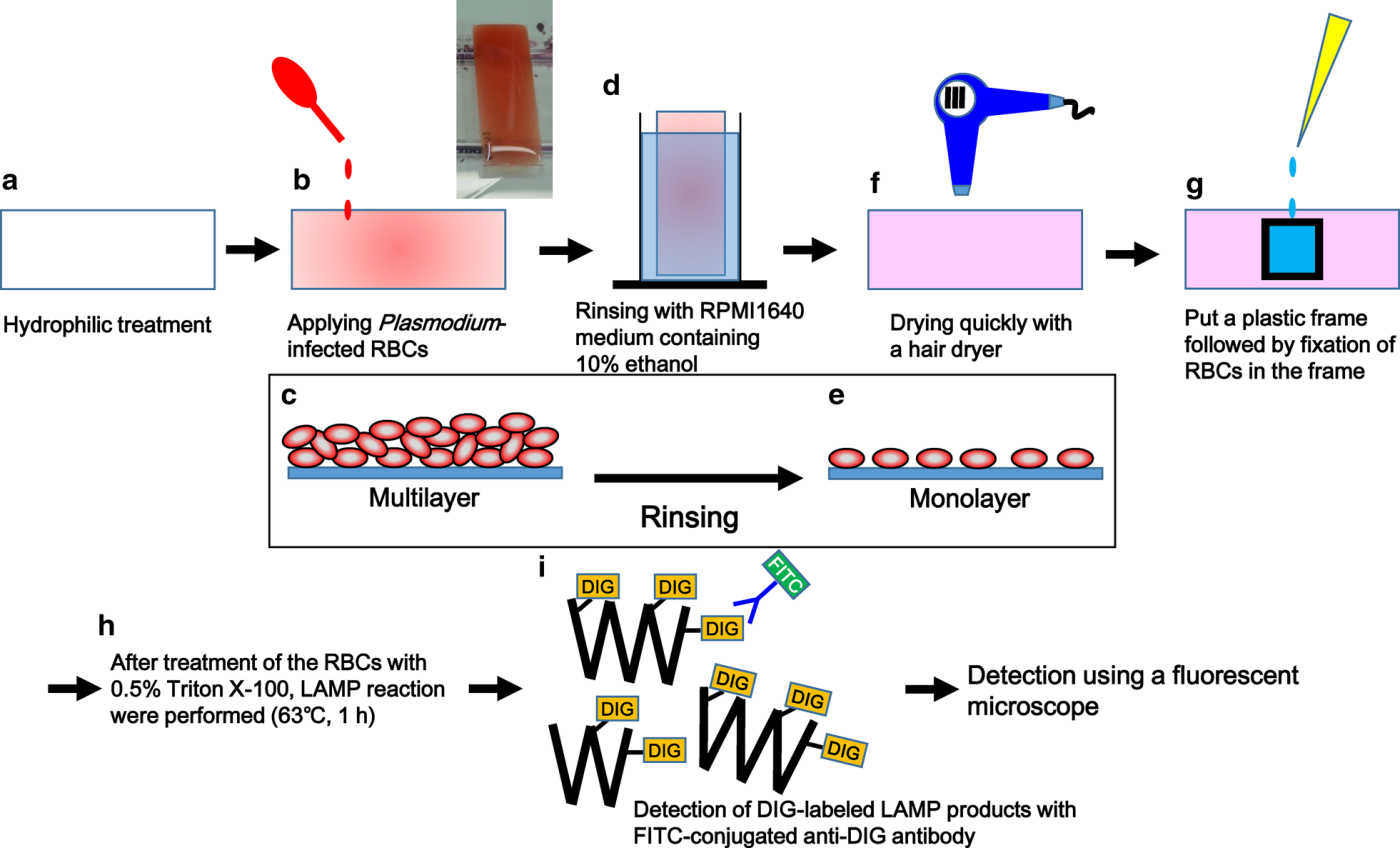
Protocol for in situ LAMP for *Plasmodium*-infected red blood cells on hydrophilic-treated COC plates. After hydrophilic treatment of COC plates (**a**), iRBCs were added to the plates, followed by 10 min standing to allow the RBCs to settle down on the plate surface (**b**). A picture of a plate (box). Schematic cross-sectional image of the RBCs forming a multilayer on a COC plate (**c**). After removing non-adherent cells (**d**), cells were spread as a monolayer on the plates (**e**). Plates were dried rapidly with a hair dryer (**f**). The cells were fixed with formalin (**g**), followed by permeabilization with Triton X-100. Then, LAMP reaction with DIG-labeled dUTP and a specific primer set for *P. falciparum 18S rRNA* gene was performed (**h**). The resulting LAMP products in the cells were visualized with FITC-conjugated anti-DIG antibody using a fluorescence microscope (**i**)

Technical details of in situ LAMP assay are described in Fig. 1. Briefly, fresh RBCs from a healthy donor with blood type O (Japanese Red Cross Society, Tokyo, Japan) were infected with *P. falciparum*, which contained more than 60% of ring-stage, more than 20% of late trophozoite-stage, and less than 20% of schizont-stage parasites. The monolayer of infected-RBCs was allowed to dry on the hydrophilic-treated COC plate (Fig. 1a–f), fixed with 20% formalin for 20 min, and then permeabilized with 0.5% Triton X-100 for 10 min. For in situ LAMP reaction, cells were incubated with LAMP reaction mixture at 63 °C on a hot plate (HHP-140D, AS ONE Corporation, Osaka, Japan) for 1 h. LAMP reaction mixture (100 µL) contained 50 µL of 2× reaction mix (Loop DNA amplification Kit, Eiken Chemical Co. Ltd., Tochigi, Japan), 4 µL of *Bst* DNA polymerase (Eiken Chemical Co. Ltd.), 3 µL of 1 mM digoxigenin (DIG)-11-dUTP (Roche, Mannheim, Germany), 5.2 µL of primer mix specific to *18S rRNA* gene of *P. falciparum* [13] containing 160 pmol of each inner primer (FIP and BIP), 80 pmol of each loop primer (FLP and BLP), and 20 pmol of each outer primer (F3 and B3), and 37.8 µL of Milli-Q water. After washing the cells with PBS, 100 µL of the diluted anti-DIG-fluorescein, Fab fragments (Roche) (1:100 in PBS), was added to the cells and incubated for 1 h at room temperature (23–28 °C). After washing with PBS, cells were nuclear-stained and mounted using SlowFade™ Gold Antifade Mountant with DAPI (Thermo Fisher Scientific Inc., Waltham, MA). Phase-contrast and fluorescence images of the cells were acquired using a fluorescence microscope (DM6000 B, Leica Microsystems, Wetzlar, Germany) with a digital camera (DFC350 FX, Leica Microsystems).

## Results

Figure 1 shows a protocol for in situ LAMP assay in iRBCs. Reactive ion-etching treatment was performed on glass-slide shaped cyclic olefin copolymer (COC) plates (Fig. 1a). Three mL of iRBCs diluted to 1% of haematocrit with RPMI 1640 medium was dropped on hydrophilic-treated plates (Fig. 1b). Plates were left undisturbed for 10 min to allow the RBCs to settle and adhere onto the plates (Fig. 1c). Non-adherent RBCs were removed by rinsing the plates for 10 s with RPMI 1640 medium containing 10% ethanol (v/v), which facilitates drying of RBCs in the next steps (Fig. 1d, e). The monolayer, which covered the entire plate, was immediately dried using a hair dryer (Fig. 1f) to avoid disrupting the morphology of the RBCs or parasites.

A plastic frame(s) (1 cm length × 1 cm width × 0.5 cm height) was glued on the plate with double-sided tape and manicure, followed by fixation of the cells with 100 µL of 20% (v/v) formalin diluted with Milli-Q water for 20 min in the frame (Fig. 1g). After removal of the fixing solution, cells were treated with 100 µL of Triton X-100 diluted with Milli-Q water [0.5% (v/v)] for 10 min (Fig. 1h). *Plasmodium* parasitizes in a parasitophorous vacuole (PV) within RBCs [23], and the treatment of iRBCs with Triton X-100 may permeabilize the PV membrane. Recently, it has been reported that the sensitivity of LAMP increased 100-fold after Triton X-100 treatment of iRBCs [24]. After removal of the solution, 100 µL of the LAMP reaction mixture containing a primer set specific for amplification of *P. falciparum 18S rRNA* gene was added to the cells (Fig. 1h). To avoid evaporation of the reaction mixture, 180 µL of mineral oil was overlaid. DIG-11-dUTP was added to the LAMP reaction mixture (30 µM at the final concentration). The plate was heated on a hot plate for 1 h at 63 °C. LAMP reaction resulted in DIG-labeled *18S rRNA* gene amplification in iRBCs (Fig. 1i). The reaction product was visualized using anti-DIG antibodies conjugated with fluorescein (FITC) by fluorescence microscopy.

**Fig. 2:**
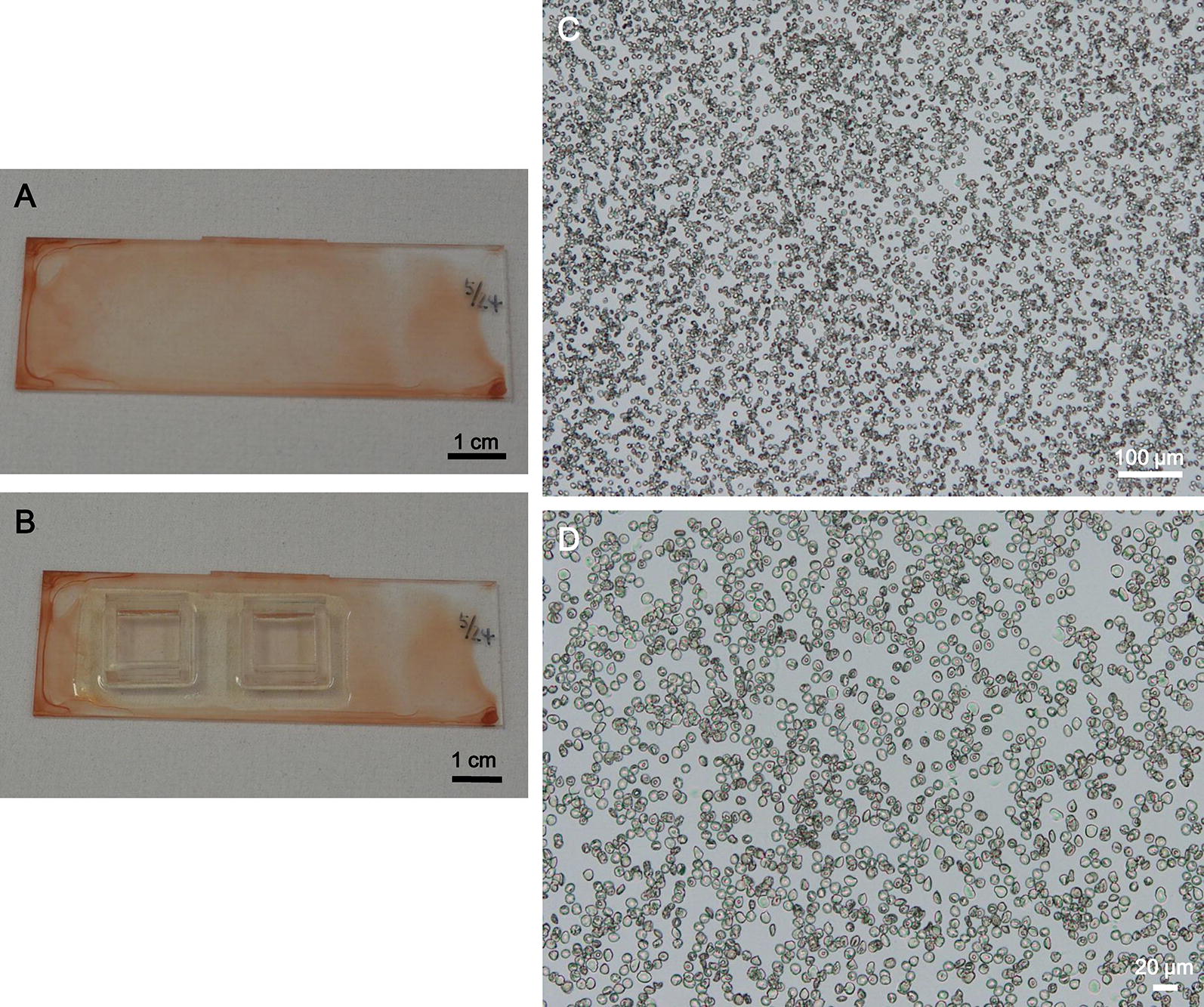
Representative images of red blood cells on hydrophilic-treated plates for in situ LAMP assay. **a**, **b** A hydrophilic-treated COC plate with dried RBCs without a plastic frame (**a**) and with plastic frames (**b**). **c**, **d** Representative microscopic images of the RBCs on the plate at ×50 magnification (**c**) and ×200 magnification (**d**)

Figure 2a shows the hydrophilic-treated COC plate after application of RBCs followed by quick drying. RBCs were uniformly spread across the plate. Figure 2b shows the plate with two plastic frames for in situ LAMP assay. When the plates were observed under a microscope, a monolayer of non-aggregated RBCs was visualized over the plate surface (Fig. 2c, d).

**Fig. 3:**
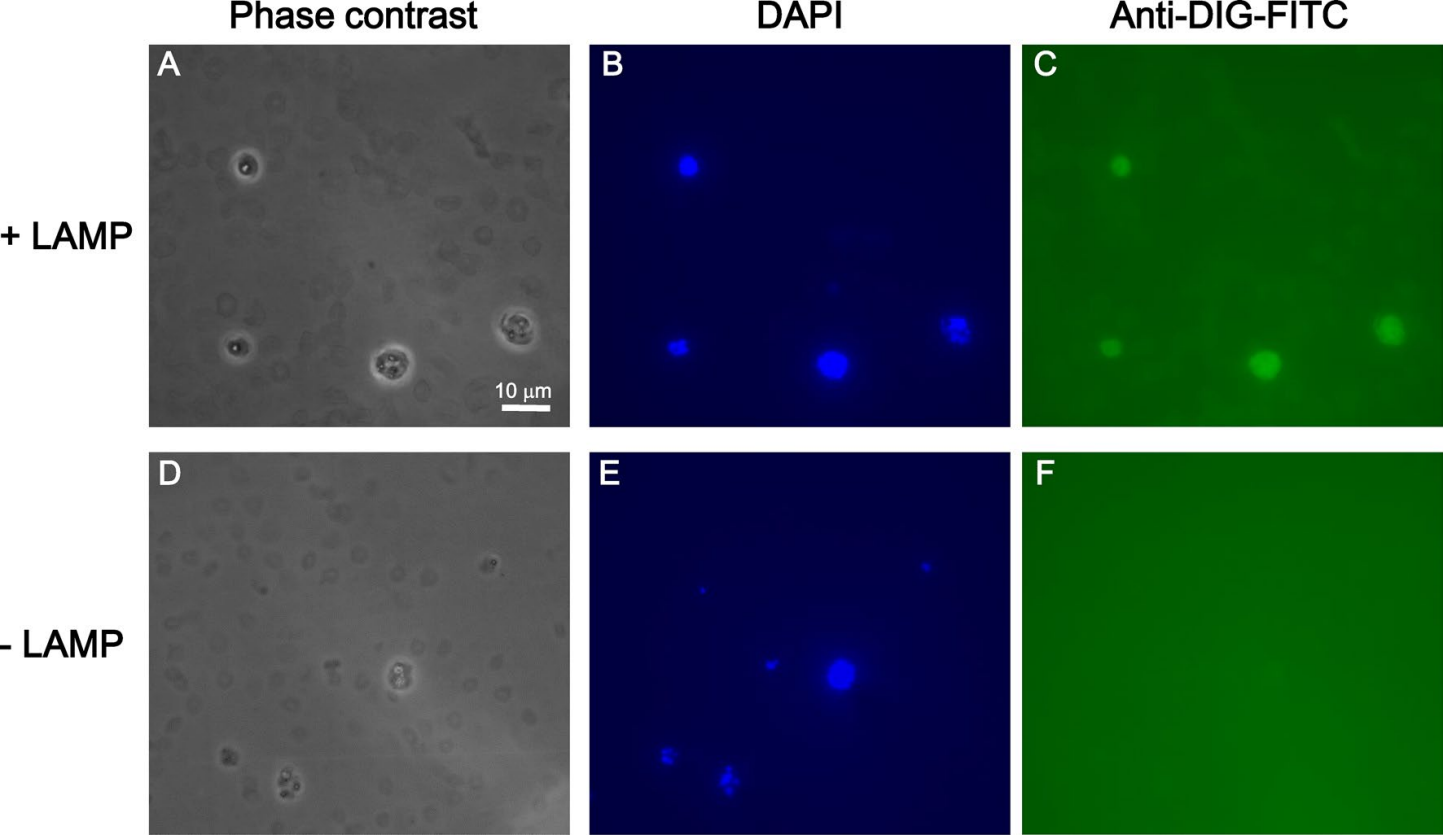
Detection of *Plasmodium falciparum* in red blood cells using in situ LAMP with DIG-labelled dUTP. **a**–**c** iRBCs on the hydrophilic-treated plates were analysed by in situ LAMP assay. Representative phase-contrast microscopic images (1000×, oil immersion) of the cells (**a**), fluorescence microscopy image in which the nucleic acid was stained with DAPI (**b**) and in which LAMP reaction was performed (**c**). **d**–**f** For a negative control, cells on the plates were handled according to the protocol described in Fig. 1, except PBS was added instead of the LAMP reaction mixture. Representative phase-contrast microscopic image of the cells (**d**), fluorescent microscopic image in which nucleic acid was stained with DAPI (**e**), and fluorescent microscopic image of cells visualized with a filter for FITC (**f**). Note that DAPI-positive RBCs are parasite-infected RBCs

Representative images after in situ LAMP assay are shown (Fig. 3a–c). For a negative control, cells were treated with Milli-Q water instead of the LAMP reaction mixture followed by detection with the FITC-conjugated anti-DIG antibody (Fig. 3d–f). To identify the parasites, iRBCs were also stained with nuclear stain 4′,6-diamidino-2-phenylindole (DAPI) (Fig. 3b, e). In this experimental condition, iRBCs were clearly observed in phase-contrast images (Fig. 3a, d) as well as by fluorescence microscopy (iRBCs stained with DAPI) regardless of the presence or absence of the LAMP reaction. While fluorescent signals in iRBCs were detected by in situ LAMP reaction (Fig. 3c), they were not detected in the negative control (Fig. 3f). These results indicate that in situ LAMP was successfully performed in iRBCs.

## Discussion

In this study, a method called in situ LAMP assay that involves forming a monolayer of iRBCs over the entire surface of hydrophilic-treated COC plates was described. This assay enables researchers to identify parasites under a microscope from a wide range of blood smears and perform genetic analysis of the parasites at a single cell level. In situ LAMP assay can be performed in a short period of 2.5 h. Considering that DNA purification is required for the standard LAMP assay, the in situ LAMP assay is rapid, easy, and inexpensive, as a COC plate is of low-cost (0.3 USD), thereby providing several advantages over the standard assay. In this study, in situ LAMP was performed in an area of 1 cm^2^ using 100 µL of the LAMP reaction mixture to acquire stable results. About four-fold increase in the amount of reagent was required as compared to the standard LAMP assay in a tube. Since FITC-conjugated anti-DIG antibody is required for in situ LAMP assay, it costs about 15 USD for one assay, and the cost also depends on the assay area and the price of the reagents used for the assay.

In situ LAMP assay could be performed on a thin blood smear prepared on glass slides. Consequently, in situ LAMP assay may be performed on blood smears prepared in field settings. Since the effect of hydrophilic-treatment on COC plates lasts for more than 2 weeks, the hydrophilic-treated plates can be taken to the field. The number of RBCs that can be analysed in COC plates is higher compared to a thin blood smear; therefore, in situ LAMP assay with hydrophilic-treated COC plates should be performed for better results. Especially, when the five species of the parasites need to be distinguished, five LAMP reaction mixtures containing the specific primer sets for each species must be prepared. In this case, since five plastic frames for the assay can be glued on the RBC monolayer on a single hydrophilic-treated plate, the COC plates are more appropriate than glass slides. The protocol for in situ LAMP assay will remain the same among the parasite species is anticipated, but this has to be verified in the future.

As microscopic analysis of Giemsa staining is commonly used for the identification of the parasite species, and whether in situ LAMP assay could be performed after Giemsa staining was investigated. iRBCs spread in a monolayer over the entire surface of hydrophilic-treated plastic plates were fixed with methanol followed by Giemsa staining. Then, in situ LAMP assay was performed using the Giemsa-stained RBC monolayer. The dye for Giemsa staining was removed during in situ LAMP assay. After in situ LAMP reaction, no FITC-labeled iRBCs that were stained with DAPI were detected. Due to methanol fixation of iRBCs, membrane of RBCs and/or PVs might not have been permeabilized by Triton X-100 treatment, or the fixed genome DNA could not serve as the template for the in situ LAMP reaction.

A fluorescent microscope is required for the in situ LAMP assay. In field settings, an optical microscope is better than a fluorescent microscope. Using 5-bromo-4-chloro-3-indolyl phosphate/nitro blue tetrazolium as the substrate for immunodetection was attempted in in situ LAMP assay with alkaline phosphatase-conjugated anti-DIG antibody instead of FITC-conjugated anti-DIG antibody. However, it was very difficult to distinguish it from the reaction product from haemozoin. On the other hand, more than 100 iRBCs were analysed by in situ LAMP assay using a fluorescent microscope, and the FITC signals were detected in all iRBCs. Further, since the specificity of anti-DIG antibody is very high, false fluorescent signals were not detected. Taken together, the specificity of in situ LAMP assay developed in this study was very high. Development of in situ LAMP assay that does not require a fluorescent microscope is important for the assay to be more widely used.

Previously, a method for automated determination of infection rate on the hydrophilic-treated COC plate was reported [5]. iRBCs stained with SYTO21, a cell-permeant green fluorescent dye, are added to the plate, allowing formation of a wide range of monolayers, consisting of more than 1.8 × 10^7^ RBCs. The number of RBCs and parasites can be calculated automatically using software tools. Thus, not only identification of the species, but also highly sensitive and quantitative detection of the parasites can be performed on the hydrophilic-treated plate. On the other hand, for the treatment of malaria, determination of the resistance of the parasites to drugs is also important. Recently, Chuhar et al. reported that LAMP can be used for the detection of the chloroquine-resistance gene of *P. falciparum* [25], suggesting the potential of LAMP assay for analysis of various drug resistance genes. Therefore, in situ LAMP can be used not only for the identification of the parasite species, but also for the detection of resistance gene of the parasite to drugs at cellular levels. Shah et al. developed a fluorescence in situ hybridization (FISH) method for the detection of iRBCs [26], and identification of the parasite species can also be possible with this method. One of the disadvantages of FISH is that if the target genes are not abundantly present (e.g., single copy gene), they cannot be detected. Since target genes can be amplified by LAMP with high sensitivity, accurate identification of parasite species and drug-resistant mutations may be possible by this method.

## Conclusions

Detection of *Plasmodium* in blood samples by microscopic examination is cardinal for definite diagnosis of malaria. Identification of the parasite species are also important for proper treatment. The technicians have to get skillful for identification of the parasite species by Giemsa microscopy. To avoid misdiagnosis, diagnosis by molecular methods should be performed especially in the difficult cases (i.e., patients with low parasitaemia, mixed infection, or receiving treatment). In situ LAMP with hydrophilic-treated COC plates enable the technicians not only to examine a number of iRBCs compared to canonical blood smears, but also to identify the parasite species by the molecular method at cellular level. Therefore, in situ LAMP may significantly improve present gold standard method for malaria diagnosis.

## Abbreviations

COC: cyclic olefin copolymer
DIG: digoxigenin
FISH: fluorescence in situ hybridization
LAMP: loop-mediated isothermal amplification
PCR: polymerase chain reaction
RBC: red blood cells
iRBC: *Plasmodium*-infected RBC
RDT: rapid diagnostic test
PV: parasitophorous vacuole

## References

[1] Fleischer B (2004). 100 years ago: Giemsa’s solution for staining of plasmodia. Trop Med Int Health.

[2] Bejon P, Andrews L, Hunt-Cooke A, Sanderson F, Gilbert SC, Hill AV (2006). Thick blood film examination for *Plasmodium falciparum* malaria has reduced sensitivity and underestimates parasite density. Malar J.

[3] Moody A (2002). Rapid diagnostic tests for malaria parasites. Clin Microbiol Rev.

[4] Kasetsirikul S, Buranapong J, Srituravanich W, Kaewthamasorn M, Pimpin A (2016). The development of malaria diagnostic techniques: a review of the approaches with focus on dielectrophoretic and magnetophoretic methods. Malar J.

[5] Hashimoto M, Yatsushiro S, Yamamura S, Tanaka M, Sakamoto H, Ido Y (2017). Hydrophilic-treated plastic plates for wide-range analysis of Giemsa-stained red blood cells and automated Plasmodium infection rate counting. Malar J.

[6] Wongsrichanalai C, Barcus MJ, Muth S, Sutamihardja A, Wernsdorfer WH (2007). A review of malaria diagnostic tools: microscopy and rapid diagnostic test (RDT). Am J Trop Med Hyg.

[7] Hänscheid T (2003). Current strategies to avoid misdiagnosis of malaria. Clin Microbiol Infect.

[8] Payne D (1988). Use and limitations of light microscopy for diagnosing malaria at the primary health care level. Bull World Health Organ.

[9] Johnston SP, Pieniazek NJ, Xayavong MV, Slemenda SB, Wilkins PP, da Silva AJ (2006). PCR as a confirmatory technique for laboratory diagnosis of malaria. J Clin Microbiol.

[10] Morassin B, Fabre R, Berry A, Magnaval JF (2002). One year’s experience with the polymerase chain reaction as a routine method for the diagnosis of imported malaria. Am J Trop Med Hyg.

[11] Ariey F, Witkowski B, Amaratunga C, Beghain J, Langlois AC, Khim N (2014). A molecular marker of artemisinin-resistant *Plasmodium falciparum* malaria. Nature.

[12] Chen D, Mauk M, Qiu X, Liu C, Kim J, Ramprasad S (2010). An integrated, self-contained microfluidic cassette for isolation, amplification, and detection of nucleic acids. Biomed Microdevices.

[13] Lau YL, Lai MY, Fong MY, Jelip J, Mahmud R (2016). Loop-mediated isothermal amplification assay for identification of five human Plasmodium species in Malaysia. Am J Trop Med Hyg.

[14] Tiono AB, Diarra A, Sanon S, Nébié I, Konaté AT, Pagnoni F (2013). Low specificity of a malaria rapid diagnostic test during an integrated community case management trial. Infect Dis Ther.

[15] Aregawi M, Cibulskis RE, Otten M, Williams R, World Health Organization, WHO Global Malaria Programme (2009). Surveillance monitoring and evaluation unit.: world malaria report 2009.

[16] Ocker R, Prompunjai Y, Chutipongvivate S, Karanis P (2016). Malaria diagnosis by Loop-mediated isothermal amplification (Lamp) in Thailand. Rev Inst Med Trop Sao Paulo.

[17] Noordhoek GT, Kolk AH, Bjune G, Catty D, Dale JW, Fine PE (1994). Sensitivity and specificity of PCR for detection of *Mycobacterium tuberculosis*: a blind comparison study among seven laboratories. J Clin Microbiol.

[18] Liu Y, Chuang CK, Chen WJ (2009). In situ reverse-transcription loop-mediated isothermal amplification (in situ RT-LAMP) for detection of Japanese encephalitis viral RNA in host cells. J Clin Virol.

[19] Maruyama F, Kenzaka T, Yamaguchi N, Tani K, Nasu M (2003). Detection of bacteria carrying the stx_2_ gene by in situ loop-mediated isothermal amplification. Appl Environ Microbiol.

[20] Wang L, Shi L, Su J, Ye Y, Zhong Q (2013). Detection of Vibrio parahaemolyticus in food samples using in situ loop-mediated isothermal amplification method. Gene.

[21] Yatsushiro S, Yamamura S, Yamaguchi Y, Shinohara Y, Tamiya E, Horii T (2010). Rapid and highly sensitive detection of malaria-infected erythrocytes using a cell microarray chip. PLoS ONE.

[22] Yamamura S, Yatsushiro S, Yamaguchi Y, Abe K, Shinohara Y, Tamiya E (2012). Accurate detection of carcinoma cells by use of a cell microarray chip. PLoS ONE.

[23] Glushakova S, Yin D, Li T, Zimmerberg J (2005). Membrane transformation during malaria parasite release from human red blood cells. Curr Biol.

[24] Hashimoto M, Sakamoto H, Ido Y, Yatsushiro S, Kajimoto K, Tanaka M (2017). Sensitivity of loop-mediated isothermal amplification increases 100-fold after Triton™ X-100 treatment of Plasmodium-infected erythrocytes. SOJ Microbiol Infect Dis..

[25] Chahar M, Mishra N, Anvikar A, Dixit R, Valecha N (2017). Establishment and application of a novel isothermal amplification assay for rapid detection of chloroquine resistance (K76T) in *Plasmodium falciparum*. Sci Rep.

[26] Shah J, Mark O, Weltman H, Barcelo N, Lo W, Wronska D (2015). Fluorescence in situ hybridization (FISH) assays for diagnosing malaria in endemic areas. PLoS ONE.

